# Characterization of Mannose-Rich Exopolysaccharides from Kefir Lactic Acid Bacteria and Their Techno-Functional Potential in Fermented Milk

**DOI:** 10.3390/foods15081322

**Published:** 2026-04-10

**Authors:** Tingting Zhang, Yunyan Li, Jingjing Leng, Zi Ye, Zhufang Duan, Bingfang Huang, Chunqiu Zhang, Muhammad Imran, Muhammad Azam, Bohan Sun, Yanglei Yi

**Affiliations:** 1 College of Food Science and Engineering, Northwest A&F University, Yangling 712100, China; zhangtt@nwafu.edu.cn (T.Z.); klynma@163.com (Y.L.); lengjj@nwafu.edu.cn (J.L.); yz428052@163.com (Z.Y.); dzf20010402@163.com (Z.D.); bingfang_h@outlook.com (B.H.); cqz@nwafu.edu.cn (C.Z.); imranbhatti973@gmail.com (M.I.); 2 National Institute of Food Science and Technology, University of Agriculture Faisalabad, Faisalabad 38000, Pakistan; muhammadazamfst@gmail.com; 3 Northwest A&F University Shenzhen Research Institute, Shenzhen 518000, China

**Keywords:** kefir, exopolysaccharides, lactic acid bacteria, fermented milk

## Abstract

Kefir grains are a valuable source of exopolysaccharide (EPS)-producing lactic acid bacteria (LAB) with potential applications in fermented dairy products. In this study, LAB isolated from kefir grains originating from five regions were screened for EPS production and probiotic-related properties. Three strains, *Lactiplantibacillus plantarum* XZ61, *Lactobacillus kefiranofaciens* EG10, and *Lentilactobacillus kefiri* EG12, were selected based on high EPS yield, antimicrobial activity, antioxidant capacity, and tolerance to acidic and bile salt conditions. After optimization, the highest EPS yield (539.57 μg/mL) was obtained from strain EG10.The purified EPS consisted of two molecular weight fractions (≈1.4 and 23~25 kDa) and was rich in mannose (33.38~61.58%). Among the three EPS, EG10-EPS exhibited superior emulsifying and flocculating properties comparable to commercial stabilizers, as well as notable ABTS^•+^ and hydroxyl radical scavenging activities. Furthermore, co-fermentation of *L. kefiranofaciens* EG10 with conventional yogurt starter cultures significantly improved exopolysaccharide content, water-holding capacity, texture, and antioxidant activity of fermented milk, particularly in cow milk. These results demonstrate the potential of kefir-derived EPS-producing LAB as natural functional cultures for fermented dairy applications.

## 1. Introduction

Exopolysaccharides (EPS) are extracellular biopolymers produced by various microorganisms, including bacteria, fungi, and cyanobacteria, during their metabolic processes [1]. A wide range of microorganisms are capable of producing EPS, among which lactic acid bacteria (LAB) are recognized as Generally Regarded as Safe (GRAS) by the World Health Organization. In recent years, EPS synthesized by LAB have attracted considerable attention due to their unique physicochemical properties and diverse biological activities. Laboratory-produced EPS have been widely applied in the food, pharmaceutical, and biotechnological fields, as well as in bioflocculant production [2]. In the food industry, LAB-derived EPS are often used as natural alternatives to commercial stabilizers because of their thickening, emulsifying, and gelling properties, which can improve the rheological behavior and sensory characteristics of fermented products [3].

In addition to their techno-functional roles, numerous studies have demonstrated that certain EPS produced by LAB can act as postbiotics, exhibiting health-promoting activities such as immunomodulatory, antitumor, antibiofilm, antioxidant, and cholesterol-lowering effects. These bioactive functions suggest that LAB-derived EPS may contribute to the promotion of human health and the prevention of chronic diseases [4,5].

Kefir, a traditional fermented dairy beverage produced through the symbiotic fermentation of diverse microorganisms, represents a rich and dynamic ecosystem containing *Lactobacillus*, *Lactococcus*, *Leuconostoc*, *Acetobacter*, and yeasts such as *Kluyveromyces marxianus*. LAB are typically the dominant members of kefir grains and play a crucial role in determining the flavor, texture, and health benefits of the final fermented product [6]. The complex microbial interactions and ecological succession occurring during kefir fermentation facilitate the natural selection of robust and functional LAB strains. Therefore, kefir provides an ideal niche for isolating and identifying EPS-producing LAB with desirable technological and biological properties, underscoring its value as a natural reservoir for developing functional starter cultures and health-promoting food ingredients [7].

In this study, a diverse group of LAB from five different sources of kefir grains was isolated and characterized. Strains exhibiting the highest EPS production were selected and screened for probiotic potential. The most promising isolates were subsequently subjected to EPS yield optimization. The extracted EPS were purified and comprehensively characterized using UV-visible spectroscopy, Fourier-transform infrared (FT-IR) spectroscopy, and high-performance liquid chromatography (HPLC) to elucidate their molecular composition and structural features. Additionally, some of the key physical properties—such as emulsifying capacity, flocculating ability, and antioxidant activities—were assessed to identify the potential of these EPS from natural sources as functional ingredients for the food and related industries.

## 2. Materials and Methods

### 2.1. Kefir Generation Fermentation

Five kefir grain samples were collected from Baotou, China (BT), Altai, Russia (EH), Tibet, China (XZ), Bremen, Germany (DG), and the North Caucasus, Russia (EG), respectively. All kefir grains were obtained from local household fermentations or artisanal producers, where they had been traditionally maintained through continuous back-slopping. Actively fermenting kefir grains were aseptically collected, transferred into sterile containers, and transported to the laboratory under refrigerated conditions. The sampling distribution (four samples from Asia and one from Europe) was primarily determined by sample accessibility and the availability of traditionally maintained kefir grains from different regions. Notably, a sample from the North Caucasus region was included as a reference due to its close association with the recognized origin of kefir. Although kefir is generally recognized to have originated from the Caucasus region, samples from different geographical locations were included in this study to reflect the diversity shaped by regional propagation practices. All samples were transported under cold-chain conditions. Prior to analysis, all samples were maintained as fresh, active kefir grains without any drying treatment. The samples were stored at −80 °C in the laboratory. After activating, the kefir grain samples were inoculated into 100 mL of sterile cow and goat milk at 25 °C at a 5% (*w*/*v*) ratio. The cow milk and goat milk were purchased from Inner Mongolia Mengniu Dairy (Group) Co., Ltd. (Inner Mongolia, Hohhot, China) and Tianjin Shepherd Dairy Co., Ltd. (Tianjin, China), respectively. The cultures were then cultivated for seven consecutive generations, during which the substrate was changed every 24 h. The EPS content, kefir grain mass, and pH of each generation of kefir were measured.

### 2.2. Isolation of LAB

Samples from the culture showing the highest EPS production were serially diluted, and 100 μL of each dilution was spread onto De Man, Rogosa, and Sharpe (MRS) agar plates (Thermo, Rockford, IL, USA). After incubation, single colonies were picked for streaking, and for preservation in MRS broth (Thermo, Rockford, IL, USA) for 24 h. After the activation of the strains, the lactic acid bacteria (LAB) were screened by Gram staining, followed by microscopic observation of their morphology.

### 2.3. Screening of High-Yielding EPS LAB

The LAB were activated and streaked on MRS agar plates for 24 h. Colonies were gently touched with a sterile toothpick and withdrawn to assess thread formation; isolates were preliminarily screened for high EPS production based on thread length [8]. The selected LAB were inoculated in MRS broth for 48 h. EPS production was determined by the phenol-sulfuric acid method [9].

### 2.4. Molecular Characterization of High EPS Producing LAB

The genomic DNA of LAB with high EPS production was first extracted by cetyltrimethylammonium bromide (CTAB). The genera of the strains were identified by RAPD-PCR based on Adesulu’s method [10] with slight modifications. The sequences of the primers were M13 (5′-GAGGGTGGCGGTTCT-3′), M14 (5′-GAGGGTGGGGCCGTT-3′) and D8635 (5′-GAGCGGCCAAAGGGAGCAGAC-3′). RAPD-PCR was performed using these primers with an annealing temperature of 42 °C for 30 s, and the products were detected using a 1% (*w*/*v*) nucleic acid gel. Strains of different species were selected for sequencing using the 16S rDNA with 27F (5′-AGAGTTTGATCCTGGCTCAG-3′) and 1492R (5′-CGGTTACCTTGTTACGACTT-3′). Sequencing results were then subjected to BLAST (https://blast.ncbi.nlm.nih.gov/Blast.cgi, accessed on 31 March 2026) in NCBI for identification.

### 2.5. Evaluation of the Probiotic Properties

Probiotic properties of LAB were determined by inoculating them into 200 μL of MRS liquid medium at a ratio of 2% (*v*/*v*), and culturing them at 37 °C for 48 h. The absorbance value reflected the growth status of the strains. The bacteriostatic properties of the strains were determined by the Oxford cup method [11]. *Escherichia coli* ATCC 25922, *Staphylococcus aureus* ATCC 25923, *Bacillus cereus* ATCC 14579, and *Listeria monocytogenes* ATCC 19115 were selected as the indicator bacteria to determine the bacteriostatic properties of the LAB. The optical density at 600 nm (OD_600_) values of lactic acid bacteria were measured after 24 h of cultivation in MRS medium at pH 2.5, pH 6.5, 0.1% (*w*/*v*) bile salt, and bile salt-free MRS medium to assess their acid and bile salt tolerance.

### 2.6. Optimization of the Yield of High-Yielding EPS LAB

The EPS production of *Lactobacillus* spp. was optimized by varying the carbon source, incubation time, pH, inoculum size, and incubation temperature. Glucose, lactose, fructose, and sucrose (Sigma-Aldrich, St. Louis, MO, USA) were selected as carbon sources, respectively. The incubation time varied across 24 h, 36 h, 48 h, 60 h, and 72 h with varying pH values (5.5, 6.0, 6.5, 7.0, and 7.5). The inoculum size was selected as 1%, 2%, 3%, 4%, and 5% (*w*/*v*), and the incubation temperature was selected as 25 °C, 30 °C, 35 °C, 40 °C, and 45 °C.

### 2.7. EPS Characterization

#### 2.7.1. EPS Extraction and Purification

*Lactobacillus* cultures were grown under the optimized conditions. Trichloroacetic acid (4%, *v*/*v*) was used to precipitate proteins at 8000 rpm, and 4 °C for 20 min. The supernatant was taken, and anhydrous ethanol was added for overnight precipitation of EPS at 10,000 rpm, 4 °C for 30 min. The supernatant was discarded, and the precipitate was reconstituted in deionized water. It was dialyzed in 8000~14000 dialysis bags at 4 °C for 48 h, and the water was changed every 4 h. The crude polysaccharide samples were obtained by vacuum freeze-drying [12].

#### 2.7.2. UV–Vis Spectral Analysis of EPS

The UV–visible spectral characteristics of EPS were analyzed using a UV–visible spectrophotometer (Agilent Cary 60, Santa Clara, CA, USA). EPS solutions (1 mg/mL) were scanned over a wavelength range of 200–800 nm at room temperature to assess their spectral profiles and evaluate sample purity. The absence of characteristic absorption peaks for proteins and nucleic acids was used as an indicator of the relatively high purity of the extracted EPS [13].

#### 2.7.3. Identification of Functional Groups and Chemical Components of EPS

Functional groups and chemical components of the EPS were analyzed using a Fourier Transform Infrared Spectrometer (FT-IR) (Bruker Optics, Ettlingen, Germany). An EPS sample (1 mg of well-dried) was mixed with 100 mg of KBr powder and thoroughly ground in a pestle mortar. In total, 30 mg of sample was pressed into tablets, which were measured by an infrared spectrometer in the range of 400 to 4000 cm^−1^ [14].

#### 2.7.4. Compositional Analysis of Monosaccharide

An EPS sample (10 mg) and 2 mol/L TFA 10 mL were placed in a sealed container and hydrolyzed for 3 h at 121 °C for reduced pressure evaporation. Then, 2 mL of distilled water was added and evaporation was repeated three times to thoroughly remove residual trifluoroacetic acid. The dried extract was reconstituted with 1 mL of ultrapure water. Then, 500 μL of 0.3 mol NaOH solution and 500 μL of 0.5 mol phenyl-methyl-pyrazolone (PMP) solution were added to the reaction tube, vortexed for 30 s and left for 90 min at 70 °C in a water bath. Afterward, 500 μL of 0.3 mol HCl and 1 mL of trichloromethane were then added, followed by vortexing for 3 min and centrifugation at 12,000 rpm for 5 min to obtain the supernatant. The supernatant was filtered using a 0.22 μm aqueous membrane to obtain the derivatized monosaccharides. The chromatographic conditions were set as follows: Agela MP-C 18 column (4.6 mm × 250 mm, 5 μm); mobile phase: phosphate solution (13.6 g KH_2_PO_4,_ 1.89 g NaOH dissolved in 1 L of distilled water): acetonitrile = 82:18; detection wavelength: 250 nm; column temperature: 30 °C; flow rate: 1.0 mL min^−1^; injection volume: 20 μL. The standards were arabinose, fucose, glucose, mannose, galactose, xylose, glucuronic, rhamnose, and D-galacturonic acid. The proportion of monosaccharides was calculated based on the peak time and peak area of each monosaccharide standard [15].

#### 2.7.5. Determination of Molecular Weight of EPS

An EPS sample (2 mg) was dissolved in 1 mL of the mobile phase and filtered using a 0.22 μm membrane. The mobile phase (0.2 mol/L NaCl-20 mmol/L, and pH 6.0 phosphate buffer) was used. The flow rate was set at 0.5 mL/min. The column temperature was set at 30 °C. A differential refractive index (DRI) detector was used for the determination of molecular weight (Mw). The set conditions were as follows: internal temperature 30 °C, and injection volume 10 μL [15].

### 2.8. Determination of the Physical Properties of EPS

#### 2.8.1. Emulsifying Properties

EPS xanthan gum, guar gum, and pectin (0.5 mg each) were dissolved in 0.5 mL of deionized water and boiled in a water bath for 30 min. After cooling, it was diluted with PBS (final volume 0.2 mL). Hexadecane (0.5 mL) was added to the mixture and vortexed for 10 min. The absorbance was measured at 540 nm (A_0_). The samples were left at 25 °C for 0, 30, 60, and 90 min, and the absorbance at 540 nm was recorded (A_t_). PBS and hexadecane were used as blanks. Emulsifying capacity was calculated using the following Equation (1) [15]:

(1) $$Emulsifying\;capacity\%=A_{t}-A_{0}\times 100$$

#### 2.8.2. Flocculation

The suspension of 5 g/L activated carbon was used as test material. Carbon suspension (10 mL) and 0.1 mL of CaCl_2_ solution (6.8 mmol/L) were mixed. Different concentrations of EPS, xanthan gum, guar gum, and pectin were added to the mixture, which was vortexed and shaken for 5 min, and then left to stand at 25 °C for 10 min. The absorbance values were measured at 550 nm. A mixture of activated carbon suspension and CaCl_2_ solution was used as a blank control [16].

#### 2.8.3. Potential and Particle Size

EPS potential was determined using a nano-laser particle sizer (SALD-2300, Shimadzu, Kyoto, Japan). A diluted sample (1 mL) was equilibrated at 25 °C for 2 min. The sample was subjected to particle size determination in the universal analytical mode. The samples were dispersed in distilled water, with a refractive index of 1.333 for water and 1.520 for sample particles at a wavelength of 633 nm, respectively. The average particle size was obtained by measuring each sample three times.

#### 2.8.4. Differential Scanning Calorimetric Analysis (DSC) of EPS

The DSC of EPS was measured using an oscillometric scanning calorimeter (Shimadzu, Japan). The 3~5 mg lyophilized powder of EPS was sealed and analyzed in an aluminum pot. The melting point and enthalpy change were determined using an empty pot as a reference point. The sample was heated from 20 °C to 400 °C at 10 °C/min, with an air flow rate at 50 mL/min.

### 2.9. Antioxidant Activity Determination

DPPH radical scavenging rate was determined as a part of antioxidant activity. To prepare the DPPH^•^ working solution, DPPH^•^ was dissolved in anhydrous ethanol to reach a concentration of 0.1 mmol/L and kept in the dark until use. The polysaccharide sample solution (0.5 mL) at different concentrations (0.5, 1.5, 2.5, 3.5, 4.5, 5.5, and6.5 mg/mL) was pipetted, and 2 mL of DPPH^•^ (0.1 mmol/L) working solution was added. The reaction was allowed to stand at room temperature, and the absorbance value was measured at 517 nm. Water was used as a blank, and ethanol was used as a control.

The ABTS^•+^ scavenging activity of the polysaccharide was determined following a previously described method with minor modifications [17]. To prepare the ABTS^•+^ stock solution, 7.4 mmol/L of ABTS^•+^ was reacted with 2.6 mmol/L of potassium persulfate by mixing them in a specific ratio. The mixture was incubated in the dark at room temperature for 12–16 h to ensure complete radical generation. Prior to the assay, the ABTS^•+^ working solution was prepared by diluting the stock solution with 0.2 mol/L PBS (pH 7.4) until its absorbance at 734 nm reached 0.70 ± 0.02. For the measurement, 0.5 mL of polysaccharide samples at various concentrations (0.5, 1.5, 2.5, 3.5, 4.5, 5.5, and 6.5 mg/mL) were mixed with 2 mL of the ABTS^•+^ working solution. The mixture was shaken thoroughly and allowed to stand at room temperature for 6 min. The absorbance was then measured at 734 nm. Water was used as a blank, and PBS was used as a control.

The hydroxyl radical scavenging test was determined by pipetting 0.5 mL of polysaccharide sample solution at different concentrations (0.5, 1.5, 2.5, 3.5, 4.5, 5.5, and 6.5 mg/mL). Then 2 mL 6 mmol/L FeSO_4_ solution, and 200 μL 0.3% H_2_O_2_ were added to it. The mixture was mixed well and then reacted at 37 °C for 30 min, and then 0.5 mL of 6 mmol/L salicylic acid was added. Salicylic acid (0.5 mL of 6 mmol/L) was added for color development with 10 mL of water. The absorbance was taken at 510 nm. The water was used as the blank sample, and the salicylic acid solution was replaced with distilled water in the control group.

### 2.10. Preparation and Characterization of Fermented Milk

#### 2.10.1. Co-Culture and Fermented Milk Preparation

*L. kefiranofaciens* EG10 was co-cultured with conventional starter cultures (*S. thermophilus* and *L. delbrueckii* subsp. *Bulgaricus*), and their growth curves were determined. For fermented milk production, the three strains were mixed at a total inoculation level of 1 × 10^9^ CFU and inoculated into cow milk and goat milk at a fixed ratio [18]. Fermentation was performed at 43 °C until the pH reached 4.6, followed by post-fermentation at 4 °C for 24 h, resulting in fermented cow milk (M) and fermented goat milk (G). Fermented cow milk (M-NC) and fermented goat milk (G-NC) prepared using only the conventional starter cultures under the same fermentation conditions were used as control groups.

#### 2.10.2. Microbiological and Physicochemical Analyses

The total viable counts of the fermented milk samples were determined using the plate count method, while titratable acidity was measured by an acid–base titration method. Water-holding capacity (WHC) was determined by centrifuging 10 *g* of fermented milk at 8000 rpm for 30 min at 4 °C. The sediment was weighed, and the WHC was calculated as the percentage of sediment weight relative to the initial sample weight.

#### 2.10.3. Texture Profile Analysis

Texture profile analysis (TPA) of fermented milk was conducted using a texture analyzer operated in yogurt mode. An A/BE probe (45 mm diameter) was used with a test speed of 1.0 mm/s, a test depth of 30.0 mm, and the trigger force set to Auto-5 g.

#### 2.10.4. Determination of Exopolysaccharides Content and Antioxidant Activity

The exopolysaccharides (EPS) content of the fermented milk samples was determined using the phenol–sulfuric acid method. The DPPH and ABTS radical scavenging activities were measured according to 2.9.

### 2.11. Statistical Analysis

Data was analyzed by Minitab 18 (Minitab, State College, PA, USA) and the results were expressed as mean ± standard deviation. Analysis of variance (ANOVA) results with *p* < 0.05 indicating a significant difference GraphPad (8.0.2, Boston, MA, USA) and Origin (2024, Northampton, MA, USA) were used for data visualization.

## 3. Results and Discussion

### 3.1. Kefir Generational Cultures

**Figure 1:**
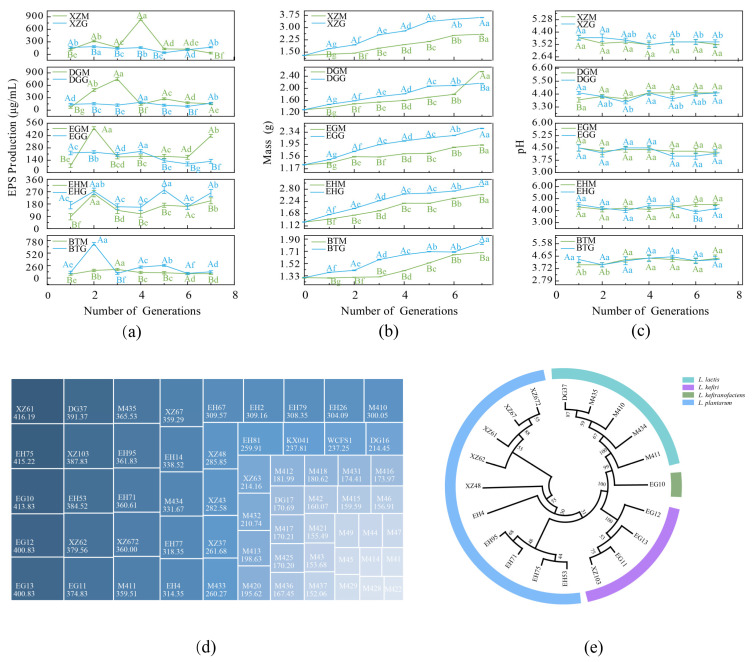
Changes in EPS production (**a**), mass (**b**) and pH (**c**) of different fermented kefir samples with grains from five sources (Different capital letters indicated that the data of the same generation of cow milk and goat milk were significantly different (*p* < 0.05), and different lowercase letters indicated that the data of different generations of the same milk source were significantly different (*p* < 0.05); EPS yield (μg/mL) (**d**) and phylogenetic analysis of 20 lactic acid bacteria isolates based on 16S rRNA gene sequences (The numbers indicate the degree of confidence) (**e**).

Activated kefir grains from five Eurasian locations were inoculated into cow’s milk and goat’s milk, respectively, to evaluate different kefir grains. The EPS content and quality were measured at each fermentation generation as shown in Figure 1. For clarity, sample abbreviations used in the figures are defined based on their geographical origin and fermentation substrate. Specifically, the first two letters indicate the sampling location (BT, EH, XZ, DG, and EG), while the suffixes “M” and “G” represent cow milk and goat milk, respectively. For example, “XZM” refers to kefir grains from Tibet (XZ) fermented in cow milk, whereas “XZG” indicates fermentation in goat milk. EPS yields differed by milk type across fermentation samples; fermented kefir samples from the BT and EH areas produced more EPS in goat’s milk, whereas the other samples produced more in cow’s milk. EPS yields also varied across fermentation generations. For fermented kefir samples from the XZ, GD, and BT areas, the yields increased at first and then decreased, peaking at the fourth, third, and second generations, respectively. By contrast, samples from the EG and EH areas showed generation-dependent fluctuations (Figure 1a). The variation in EPS content during fermentation may be the influenced by microbial-substrate adaptation. A previous study reported that changes in distinctive fatty acids, casein structures and unique medium-chain fatty acids during fermentation promoted the growth of microbiomes [19]. The fluctuation and the observed decline in EPS yield after its peak are likely due to excessive fermentation, which leads to nutrient depletion and metabolic changes that decrease EPS biosynthesis [20].

A consistent increase in mass was observed in all five kefir grain samples (Figure 1b). Notably, all grains achieved a higher mass when cultured in goat’s milk compared to cow’s milk, with the XZ sample showing the fastest increase. The pH values of the fermented kefir samples stabilized at approximately 4.5 on both milk substrates (Figure 1c). Previous studies have primarily investigated single-generation fermentation, while the results in Figure 1 suggest that kefir grains undergo adaptive changes in mass and pH values over multiple generations, which directly affect their EPS production. Kefir grains dynamically adjust their microbial communities in response to environmental conditions, leading to the observed fluctuations in EPS yield [19,20]. Therefore, identifying the specific microbial species involved in these variations, along with optimizing fermentation conditions, will be the primary focus of future research aimed at enhancing EPS yield for industrial applications.

### 3.2. Screening of High-Yielding EPS LAB

High-yielding EPS-producing LAB were isolated through MRS agar plate culturing and single colony purification. A total of 124 strains were isolated from the BT sample, 92 from XZ, 99 from EH, 40 from DG, and 14 from EG. Microscopy and Gram staining were used to preliminary identify lactobacilli, resulting in the identification of 61 strains from BT, 75 from XZ, 42 from EH, 30 from DG, and 8 from EG. The “drawing” method was employed as a sketchy qualitative technique to assess the EPS production capability of the bacterial colonies; sixty isolates that exhibited high drawing ability were then included for EPS content determination. As shown in Figure 1d, five strains produced EPS exceeding 400 μg/mL and twenty strains achieved yields greater than 300 μg/mL, thereby qualifying them as candidates for subsequent screening. These results suggested the varying EPS production capability among LAB strains in kefir grains and highlighted five strains with EPS yields over 400 μg/mL. In previous studies, EPS yields of naturally occurring kefir-derived LAB have seldom exceeded approximately 600 µg/mL under non-optimized culture conditions [21]. The yield obtained in this study (>400 µg/mL) therefore represents a relatively high production level for such naturally isolated strains. Considering the inherent limitations of non-optimized fermentation media and culture parameters in wild LAB, achieving 400 µg/mL underscores the strong potential of these isolates as high-yield EPS producers and provides a solid foundation for further process optimization and scale-up.

### 3.3. Molecular Biological Characterization of LAB

The 25 isolates were first differentiated by RAPD-PCR, and five strains with identical profiles were excluded. The remaining 20 isolates were subsequently subjected to DNA sequencing, and a phylogenetic tree was constructed (Figure 1e). The results showed that the isolates belonged to four species: five isolates were identified as *Lactococcus lactis*, eleven as *L*. *plantarum*, three as *L*. *kefiri*, and one as *L*. *kefiranofaciens*. Isolates of the same species clustered together, indicating high species-specificity and sequence similarity. Bootstrap values for the major branches were generally high, supporting the reliability of the phylogenetic classification. The 16S rRNA characterization results were uploaded to NCBI with accession numbers as follows: DG37 (PZ221242), EG10 (PZ221243), EG11 (PZ221244), EG12 (PZ221245), EG13 (PZ221246), EH4 (PZ221247), EH53 (PZ221248), EH71 (PZ221249), EH75 (PZ221250), EH95 (PZ221251), M410 (PZ221252), M411 (PZ221253), M434 (PZ221254), M435 (PZ221255), XZ48 (PZ221256), XZ61 (PZ221257), XZ62 (PZ221258), XZ67 (PZ221259), XZ103 (PZ221260), and XZ672 (PZ221261).

### 3.4. Evaluation of the Probiotic Properties of LAB with High EPS Production

**Figure 2:**
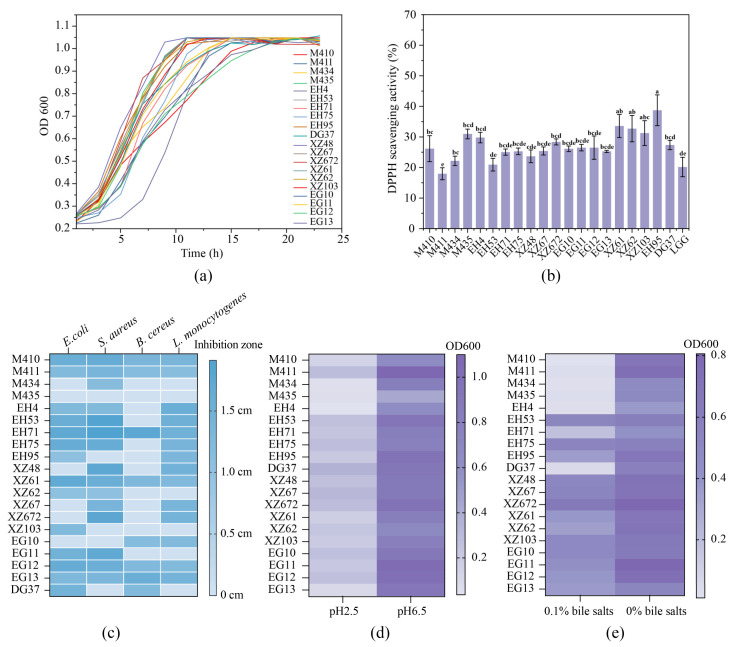
The growth curve (**a**), antioxidant capacity (**b**), bacteriostatic activity (**c**), acid resistance (**d**), and bile salt resistance (**e**). Different letters suggest significant difference (*p* < 0.05).

As high EPS-yielding strains, the potential probiotic properties of the strains were further evaluated. The growth curves of the 20 identified LAB were first determined and most of strains reached stationary phase within 15 h. Strain XZ48 took 5 h to enter the logarithmic phase, while M435 showed a longer logarithmic phase (Figure 2a). The antioxidant properties were evaluated using a DPPH^•^ assay, with the known probiotic *Lactobacillus rhamnosus* GG (LGG) used as a control. M435, EH4, EG12, XZ61, XZ62, XZ103, and EH95 exhibited high antioxidant activity (Figure 2b). The free radical scavenging and oxidative reducing properties of LAB are often attributed to the production of bioactive metabolites, particularly EPS and peptides, which can donate hydrogen atoms or electrons to neutralize reactive free radicals [22]. Our findings are consistent with this mechanism, as the strains exhibiting the highest antioxidant activity were also observed to produce substantial amounts of EPS in our cultivation assays.

The bacteriostatic properties of the LAB strains were determined against four representative pathogens: *E. coli*, *S. aureus*, *B. cereus*, and *L. monocytogenes*. Strains M410, M411, EH71, XZ61, EG12, and EG13 demonstrated effective inhibition of all four indicator bacteria (Figure 2c) suggesting their antimicrobial activity, possibly due to the high production of organic acids, bacteriocins, and hydrogen peroxide [23]. These strains with high antimicrobial activity were further evaluated under acid and bile salt condition, as probiotic bacteria usually have to survive the harsh conditions in gastrointestinal tract to exert their health benefits [24]. Strains M411, EH53, EH71, EH75, DG37, XZ67, EG10, and EG12 showed better survival at a harsh pH of 2.5 after 24 h (Figure 2d). Similarly, strains EH53, EH75, EH95, XZ67, XZ61, XZ103, EG10, EG11, EG12, and EG13 grew well under 0.1% (*w*/*v*) bile salt (Figure 2e). These results are consistent with Ming-Ju Chen et al. (2017), who indicated that acid and bile salt resistance are critical attributes for probiotic viability in the gastrointestinal tract [25]. Isolation of naturally resilient strains from traditional fermented foods represents an effective strategy for discovering robust probiotic candidates [26]. In this regard, kefir contains a complex and stable microbial consortium, harboring unique probiotic genera such as *Lactobacillus*, *Lactococcus*, *Leuconostoc*, *Acetobacter*, and yeasts, which together contribute to its distinctive flavor and potential health benefits [6]. Among these microbiomes, *L. kefiranofaciens* and *L. plantarum* possess tolerance to acidic and bile conditions, and are capable of producing bioactive EPS with antimicrobial and antioxidant properties, making them promising candidates for probiotic applications. Previous studies have isolated *L. kefiranofaciens* strains with probiotic potential from kefir based on acid and bile tolerance [25]. Moreover, in vivo and cell-based studies have demonstrated that kefir-derived LAB often exhibit notable anti-inflammatory and gut-protective effects. For instance, the EPS from *L. plantarum* YW11 was shown to modulate the composition of the gut microbiota and suppress the lipopolysaccharide (LPS)-induced production of tumor necrosis factor-α (TNF-α) and interleukin-8 (IL-8). Similarly, the EPS from *L. kefiranofaciens* subsp. *kefiranofaciens* ATCC 43761 alleviated colitis in mice by regulating the NF-κB signaling pathway and enhancing intestinal barrier integrity [27,28]. These findings further emphasize kefir as a valuable reservoir of functional LAB strains with both technological and health-promoting potential.

Therefore, based on the taxonomic distribution of the isolates, one representative strain was selected from each of the three rod-shaped species for further study, while *L*. *lactis* strains were not included due to their relatively limited role in kefir-specific EPS production. The selection was further supported by their relatively higher EPS yield, together with antimicrobial activity, antioxidant activity, acid tolerance, and bile salt tolerance. The three selected strains were *Lactiplantibacillus plantarum*, *Lactobacillus kefiranofaciens*, and *Lentilactobacillus kefiri*, designated as XZ61, EG10, and EG12, respectively.

### 3.5. Optimization of the EPS Yield of the Three LAB

**Figure 3:**
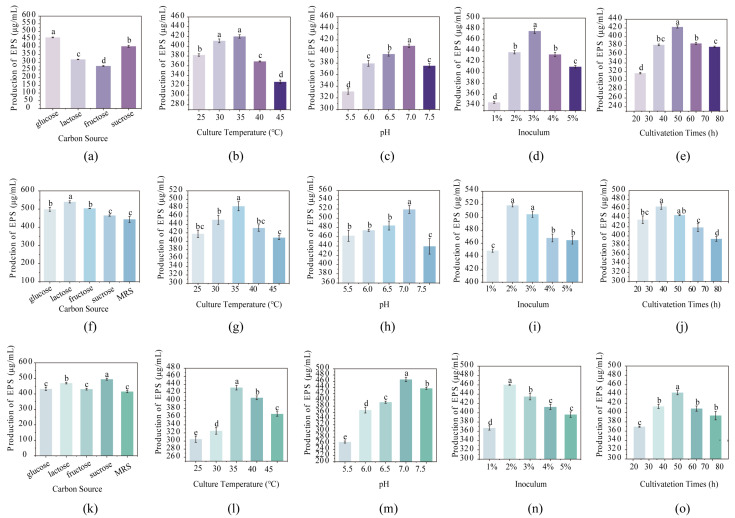
The production of EPS by XZ61 (**a**–**e**)/EG10 (**f**–**j**)/EG12 (**k**–**o**) under different carbon sources, culture temperatures, pH values, inoculation amounts and culture durations. Different letters suggest significant difference (*p* < 0.05).

EPS production is influenced by multiple factors during fermentation, including the carbon source, cultivation time, pH, inoculum size, and cultivation temperature [29]. Therefore, EPS production for each of the three selected strains was optimized according to these parameters. As shown in Figure 3a,f,k, different carbon sources had varying impact on EPS yield. Glucose proved to be the optimal carbon source for strain XZ61, which yielded 461.69 μg/mL of EPS. Lactose was optimal for EG10 (539.57 μg/mL), and sucrose was best for EG12 (495.01 μg/mL). It has been reported that different carbon sources can influence the yield of EPS by modulating different metabolic pathways in LAB, which are associated with the EPS biosynthetic pathway [30].

The optimal cultivation temperature was then determined. For all three strains, 35 °C was found to be the optimal temperature (Figure 3b,g,l). Similarly, pH value of 7.0 was optimal for EPS production. (Figure 3c,h,m). Consistent with prior research, Zhao and Liang (2023) identified 34 °C and pH 6.4 as optimal for EPS production by *L. plantarum* MC5 [31]. Overall, these findings suggest that a temperature close to 35 °C and a near-neutral pH environment favor EPS biosynthesis in LAB.

Inoculum size alters cell growth dynamics, metabolic fluxes, and the culture environment, thereby affecting the rate, timing, and net accumulation of EPS [32]. As shown in Figure 3d,i,n, the optimal inoculum was 3% (*v*/*v*) for XZ61, whereas EG10 and EG12 showed the highest EPS yield at 2% (*v*/*v*). Previous research has reported that an inoculation of 2%~3% promoted EPS production during LAB fermentation. Minari et al. (2024) reported that a 2% inoculum yielded the highest EPS production for *Lactobacillus casei* Ke8 [33], and Okoro et al. (2021) reported 3% inoculum as optimal for *Rhodotorula mucilaginosa* sp. GUMS16 [34]. These findings suggest that the inoculum amount must be optimized for each strain to balance growth and product formation, since different microorganisms exhibit distinct physiological and environmental requirements during cultivation, which affect their EPS synthesis. An appropriate inoculum size is critical, since too low a seed density result in a prolonged lag phase and insufficient biomass for EPS production, whereas an excessive inoculum may lead to nutrient depletion, the accumulation of inhibitory metabolites, and thus reduced EPS yield [35].

As shown in Figure 3e,j,o, the optimal cultivation time was 48 h for XZ61 and EG12, and 36 h for EG10. A 48 h cultivation period was widely reported in previous report on EPS production by *L. rhamnosus* 519, and *Weissella cibaria* GA44, [36,37]. However, there is also research reporting 36 h as the optimum cultivation time, such as Yang et al. (2025), who demonstrated that *Lacticaseibacillus paracasei* CICC 20241 produced maximum EPS after 36 h [38]. This difference may be due to the influence of different nutrient intake patterns of each strain [39]. After optimization, the EPS yields of XZ61, EG10, and EG12 increased from approximately 420 μg/mL to 461.69, 539.57, and 495.01 μg/mL, respectively, corresponding to 1.1- to 1.3-fold improvements. The highest yield was achieved by EG10 at 539.57 μg/mL. These results indicate that rational optimization of fermentation parameters can substantially enhance EPS productivity, even in natural kefir isolates, without the need for genetic modification, thereby supporting their potential for industrial application.

### 3.6. Chemical Component of EPS

**Figure 4:**
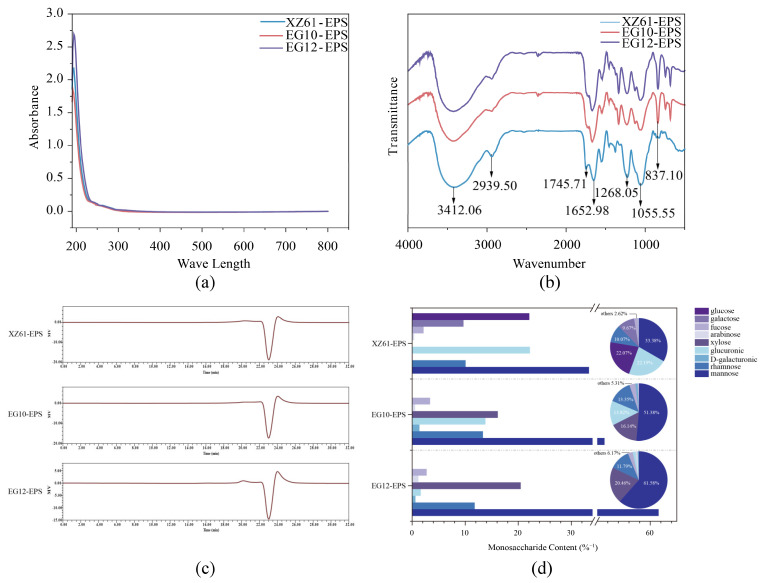
UV-vis analysis (**a**) FT-IR analysis (**b**) EPS gel permeation chromatogram (**c**) and EPS monosaccharide composition (**d**) of XZ61-EPS, EG10-EPS, EG12EPS.

To further identify the EPS component and structure, FT-IR analysis was applied. The UV-Vis spectra indicate negligible absorption at 280 nm, suggesting the presence of only trace protein contaminants in the purified EPS samples (Figure 4a). FT-IR spectroscopy was then used to characterize the functional groups of each purified EPS (Figure 4b). All samples showed a broad, significant absorption peak around 3500 cm^−1^, suggesting the characteristic hydroxyl (−OH) groups and indicative of the polysaccharide backbone structure. Notably, the XZ61-EPS spectrum displayed a more intense C−H stretching vibration at 2900 cm^−1^, suggesting a higher methylene (−CH_2_) group content compared to both EG10-EPS and EG12-EPS. The presence of absorption bands near 1600 cm^−1^ and 1700 cm^−1^, corresponding to aldehyde and carboxyl (C=O) stretching vibrations respectively indicated the presence of acidic sugars. Strong absorption bands around 1000 cm^−1^ corresponded to C−O−C stretching vibrations, confirming the presence of glycosidic linkages [40]. These results of FT-IR analysis confirmed the presence of typical functional groups in polysaccharides. The broad −OH peak at 3500 cm^−1^ and the C−O−C peaks near 1000 cm^−1^ confirmed the glycosidic linkages. The distinct C−H vibrations in XZ61-EPS indicate structural differences, which may influence its physical properties. Variations in the functional groups of acidic sugars influence structure–activity relationships, thereby affecting the biological activity and stability of EPS [41]. Jiao et al. (2010) characterized EPS from *Lactobacillus delbrueckii* ssp. *bulgaricus* SRFM-1 using FTIR and identified similar peaks for hydroxyl (3400 cm^−1^) and carboxyl (1700 cm^−1^) groups, consistent with our findings [42]. Similarly, Imran et al. (2016) reported analogous spectra for EPS produced by *L. plantarum* NTMI05 and NTMI20, with peaks at 3400 cm^−1^ and 1700 cm^−1^ [43]. Moreover, Bouzaiene et al. (2024) examined EPS from *L. plantarum* C7, which exhibited hydroxyl groups at 3278 cm^−1^ and carboxyl groups at 1620 cm^−1^; this EPS also demonstrated potent antioxidant and antibacterial properties [44].

### 3.7. Molecular Weight of EPS

The molecular weights of EPS produced by the three different strains were determined by high-performance size-exclusion chromatography (HPSEC). A standard curve was established by plotting the logarithmic molecular weights of dextran standards against their respective retention times resulting in the linear equation, y = -0.3243x + 10.953, with a coefficient of determination (R^2^) of 0.9982. Two distinct peaks were observed for each EPS, excluding the salt peak (Figure 4c). The XZ61-EPS exhibited retention times of 20.238 min and 24.079 min, corresponding to molecular weights of 23.726 kDa and 1.394 kDa, respectively. Similarly, for EG10-EPS had molecular weights of 23.726 kDa and 1.426 kDa. The molecular weights of EG12-EPS were calculated as 25.451 kDa and 1.460 kDa, respectively. Compared with previously reported LAB-derived EPS, whose molecular weights range from a few kDa to several hundred kDa [45], the EPS obtained in this study fall within the low-to-medium molecular weight range, suggesting that they possess relatively open or moderately branched molecular architectures, which may enhance molecular flexibility and facilitate interactions with reactive species or metal ions. Such relatively low molecular weights may enhance solubility and are often associated with higher bioactivity [46].

The observed variability in the molecular weights among the three EPS samples can be primarily attributed to the intrinsic genetic regulation and the specific enzymatic activities within the *eps* gene clusters of the different strains [47]. The synthesis of EPS in lactic acid bacteria is a complex process where chain length is precisely controlled by specific proteins, such as the chain length determinant and various glycosyltransferases. Differences in the expression levels or catalytic efficiencies of these enzymes among XZ61, EG10, and EG12 likely lead to the distinct degrees of polymerization observed in the HPSEC profiles. Furthermore, the presence of two distinct peaks suggests a dual-pathway biosynthetic mechanism or varying rates of chain termination relative to elongation. This variability may also be influenced by the metabolic flux of intracellular sugar nucleotide precursors, such as UDP-glucose or UDP-galactose, where slight shifts in precursor availability among the strains result in the recorded fluctuations between the high-molecular-weight fractions and the shorter oligosaccharide-like peaks [48]. Such structural diversity, dictated by the unique metabolic profile of each strain, ultimately governs the physicochemical properties and the subsequent biological activities of the resulting exopolysaccharides.

### 3.8. EPS Monosaccharide Composition

The monosaccharide composition of EPS significantly influences its structure, functionality, and properties [49]. The monosaccharide composition of the EPS produced by the three strains was characterized (Figure 4d). XZ61-EPS is a heteropolysaccharide mainly composed of mannose (33.38%), glucose (22.07%), and glucuronic acid (22.19%), with smaller amounts of rhamnose (10.07%), galactose (9.67%), D-galacturonic acid (0.09%), arabinose (0.37%), and fucose (2.16%). However, both EG10-EPS and EG12-EPS lacked glucose and galactose but were rich in mannose and xylose. Specifically, EG10-EPS consisted of mannose (51.38%), xylose (16.14%), glucuronic acid (13.82%), rhamnose (13.35%), fucose (3.38%), D-galacturonic acid (1.37%), and arabinose (0.56%). And EG12-EPS was composed of mannose (61.58%), xylose (20.46%), rhamnose (11.79%), fucose (2.74%), arabinose (1.17%), glucuronic acid (1.63%), and D-galacturonic acid (0.63%). The high glucuronic acid content in XZ61-EPS is notable, as uronic acids are well-known for their various bioactive properties, including immunomodulatory potential [50]. This may contribute to its potential health-promoting function. Xylose, present in EG10-EPS and EG12-EPS but absent in XZ61-EPS, is also commonly detected in functional heteropolysaccharides and has an impact on the solubility and prebiotic potential of EPS. The detected uronic acids in the three EPS samples may suggest their potential antioxidant and immunomodulatory functions. Overall, the monosaccharide compositions of these EPS resemble those reported for other bioactive LAB EPS, suggesting that the high mannose, glucuronic acid, and xylose contents could contribute to their potential health-promoting functions, including antioxidant and immunomodulatory activities.

### 3.9. Physical Properties of EPS

**Figure 5:**
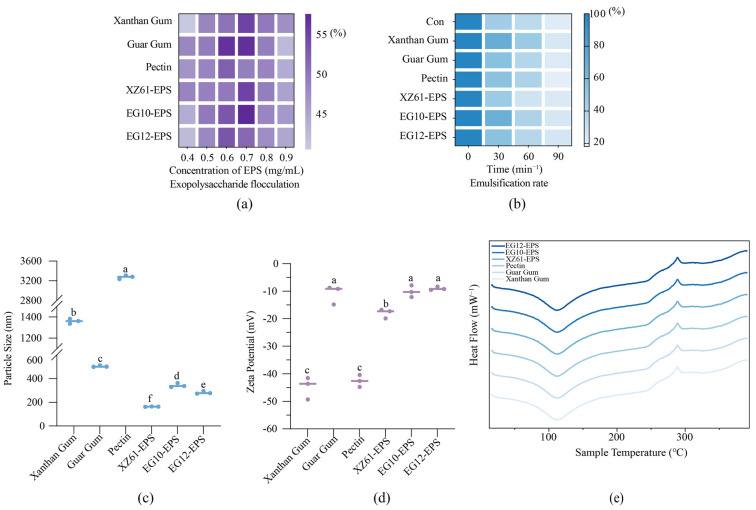
Flocculation (**a**) emulsification rate (**b**) particle size (**c**) zeta Potential (**d**) and heat flow variations (**e**) for six EPS (EG12, EG10, XZ61, Pectin, Guar Gum, Xanthan Gum). Different letters suggest significant difference (*p* < 0.05).

Flocculants, crucial in industries like wastewater treatment and food processing, are categorized as inorganic, organic synthetic, or bioflocculants. EPS, as a natural bioflocculant, have garnered significant attention due to their advantages of biodegradability and non-toxicity compared to their synthetic counterparts [51]. The flocculating properties of the EPS were tested using an activated carbon suspension (Figure 5a). Carboxylate groups in EPS chelate Ca^2+^ to form ionic bridges that promote the flocculation of activated carbon particles [52]. Higher uronic acid content (e.g., glucuronic acid) increases the density of carboxylate sites and thereby enhances flocculation. The results demonstrated that flocculating activity was directly proportional to EPS concentration. The highest flocculating activity was observed at 0.7 mg/mL for xanthan gum, guar gum, XZ61-EPS, and EG10-EPS, while EG12-EPS exhibited optimal flocculation at a slightly lower concentration of 0.6 mg/mL. This trend of increasing flocculation with concentration is consistent with findings for *L. plantarum* MM89 [53]. The variation in optimal concentrations among the EPS samples is likely attributable to differences in their molecular weight, monosaccharide composition, and overall structural features. In particular, the higher mannose content in EG12-EPS may facilitate interactions with divalent cations, resulting in more effective flocculation at slightly lower concentrations. Plant-based and microbial polysaccharides usually exhibit high emulsifying properties, ensuring widespread applications in food and pharmaceuticals [54]. Emulsifying ability is measured by the stability of the emulsion over time, typically by monitoring absorbance after 30 and 60 min. In comparison to commercial controls (xanthan gum, guar gum, and pectin), the EPS showed varying performance. Xanthan gum exhibited the best emulsifying activity, maintaining stability at 60 min (Figure 5b). The EG10-EPS performed slightly weaker emulsification than xanthan gum but better than guar gum. Kowsalya et al. (2023) reported high emulsion activity for EPS from *L. plantarum* strains in edible oils, similar to the strong emulsifying behavior of EG10-EPS [55]. Saleem et al. (2021) attributed the high emulsifying activity of EPS from *L. plantarum* S123 to its spongy, porous structure, which may be the reason why EG10-EPS outperformed XZ61-EPS [56]. Particle size and zeta potential are critical indices reflecting the stability of colloidal solutions like EPS dispersions. Smaller particle sizes enhance dispersion and reduce sedimentation, while a higher absolute zeta potential value indicates greater electrostatic repulsion between particles, preventing aggregation and improving overall system stability [57]. As shown in Figure 5c, the particle sizes of XZ61-EPS, EG10-EPS, and EG12-EPS were lower than those of the xanthan gum, guar gum, and pectin controls, indicating that the three experimental EPS samples were more easily dispersed in the system. Regarding zeta potential, all measured values were negative. The three EPS did not show significant differences from xanthan gum and pectin, suggesting their similar ability to form a stable system (Figure 5d). This stability suggests these EPSs have strong potential for use as food additives to enhance product stability. These findings of zeta potential measurements coincide with results from Balyan et al. (2024), who reported stable emulsions with a zeta potential of −32 mV for EPS from *L. plantarum* ATCC 8014 [58]. Overall, these results demonstrate that the EPS produced by *L. plantarum* XZ61, *L. kefiranofaciens* EG10, and *L. kefiri* EG12 possess desirable techno-functional properties. Such characteristics are highly valued in industrial applications, particularly in food, cosmetics, and pharmaceutical formulations where natural, biodegradable, and non-toxic stabilizers are preferred [2]. The combination of effective flocculation, emulsion stabilization, and colloidal stability indicates that these EPS serve as promising bio-based alternatives to commercial polysaccharides like xanthan and guar gums. Future studies focusing on large-scale production and rheological behavior could further validate their potential for commercial exploitation.

### 3.10. Differential Scanning Calorimetry (DSC) Analysis of EPS

EPS with greater heat resistance are considered more promising for industrial use, as high thermal stability ensures better applicability during food processing. In this study, DSC was employed to evaluate the thermal stability of the EPS samples, where glass transition and degradation temperatures are indicative of structural integrity and heat resistance. The DSC analysis from 0 °C to 400 °C revealed two distinct endothermic peaks and one exothermic peak across all six samples (guar gum, xanthan gum, pectin, EG10-EPS, EG12-EPS, and XZ61-EPS). The consistent peak at 110 °C in all samples suggested a heat absorption process (Figure 5e). This peak is associated with the loss of adsorbed water or the volatilization of residual solvent. A second, prominent exothermic peak was observed at approximately 290 °C. This peak suggested an oxidative decomposition process, driven by the interaction of the polysaccharide with atmospheric oxygen [59]. Compared to traditional polysaccharides such as guar gum and xanthan gum, XZ61-EPS, EG10-EPS, and EG12-EPS exhibit more pronounced thermal decomposition, which may provide advantages in applications that require structural modification and functional activation.

### 3.11. Antioxidant Activity

**Figure 6:**
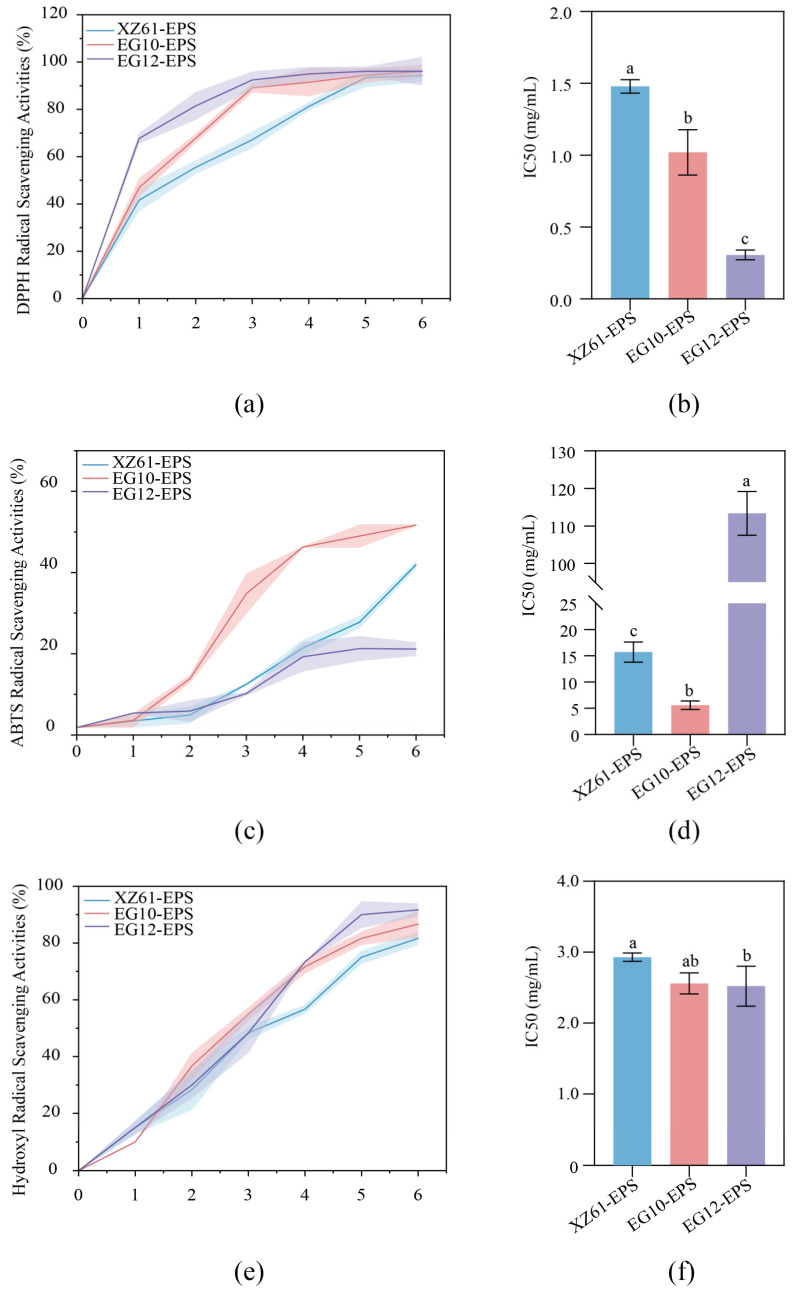
Antioxidant activity of XZ61-EPS, EG10-EPS, EG12-EPS: DPPH radical scavenging activity (**a**) and IC_50_ (**b**); ABTS radical scavenging activity (**c**) and IC_50_; (**d**); hydroxyl radical scavenging activity (**e**) and IC_50_ (**f**). Different letters suggest significant difference (*p* < 0.05).

To further assess the antioxidant potential of the EPS, three complementary assays were conducted, including DPPH radical scavenging, ABTS radical scavenging, and hydroxyl radical scavenging (Figure 6). All three EPS exhibited a concentration-dependent increase in DPPH radical scavenging activity. EG12-EPS demonstrated the strongest scavenging activity, exceeding 90% at 3 mg/mL and yielding a half-maximal inhibitory concentration (IC_50_) of 0.3 mg/mL. EG10-EPS scavenged over 90% scavenging at 5 mg/mL with an IC_50_ of 1.02 mg/mL. XZ61-EPS showed the lowest activity, only with an IC_50_ of 1.45 mg/mL (Figure 6a,b). Kowsalya et al. (2023) reported the EPS from *L. plantarum* had DPPH^•^ scavenging activities of 89.77% and 93.1% at 1 mg/mL [55]. Also, EPS from *L. kefiranofaciens* had an IC_50_ value of 0.4 mg/mL and reported a higher ABTS^•+^ scavenging activity with increase in EPS concentration. However, the ABTS^•+^ scavenging activities of the three EPS were different. EG10-EPS showed strongest ABTS^•+^ scavenging activity of 49.86% and IC_50_ of 5.12 mg/mL, while the activity of EG12-EPS decrease significantly with only 19.32% at 6 mg/mL (Figure 6c,d). This may be due to the fact that, at high concentrations, polysaccharides undergo self-entanglement, aggregation, or gelation. This may hinder their antioxidant active sites and thus reduce their free radical scavenging capacity [60]. As shown in Figure 6e,f, all three EPS samples showed concentration-dependent hydroxyl radical scavenging activity. The scavenging rates of the three EPS were relatively similar across concentrations. EG12-EPS still showed the most effective activity, which exceeding 90% at 5 mg/mL with an IC_50_ of 2.52 mg/mL. EG10-EPS had a similar IC_50_ value of 2.55 mg/mL. The strong hydroxyl radical scavenging activity of EPS involved the chelation of pro-oxidant metal ions such as Fe^2+^ and Cu^2+^, thereby inhibiting hydroxyl radical formation by the Fenton reaction [61]. These results suggest that the three isolated EPS exhibit notable antioxidant activities, which not only highlight the probiotic potential of their producing strains but also imply that such benefits are likely associated with their EPS production and the health-promoting properties of these polysaccharides. Notably, although *L. kefiri* EG12 exhibited the highest antioxidant activity among the isolates, followed by *L. kefiranofaciens* EG10 and *L. plantarum* XZ61, strain selection for further application was not based solely on a single functional parameter or EPS yield. Instead, a multi-criteria approach considering biological safety, regulatory compliance, and technological fitness was employed. *L. kefiranofaciens* EG10 was considered the most suitable candidate for practical application due to its well-established role in kefir fermentation and its recognized safety status in global food systems. This species complies with commonly accepted food-grade microbial criteria and is consistently included in the Qualified Presumption of Safety (QPS) list by EFSA and other relevant inventories of microorganisms permitted for use in food [62].

In contrast, while EG12 showed promising functional properties, the application of *L. kefiri* in standardized food systems may require more extensive safety evaluations regarding its antibiotic resistance profiles and hemolytic activity as a pure starter culture, depending on specific regional regulatory frameworks [63]. Furthermore, although XZ61is a well-recognized safe species, its relatively lower antioxidant performance in this study made it less competitive than EG10. Therefore, EG10 was selected for subsequent fermentation experiments as it represents the optimum balance between robust antioxidant efficacy and high regulatory safety. Future studies could further explore the relationship between EPS structure, antioxidant activity, and the in vivo probiotic or postbiotic effects of these specific LAB strains.

### 3.12. Quality Characteristics of Fermented Milk

**Figure 7:**
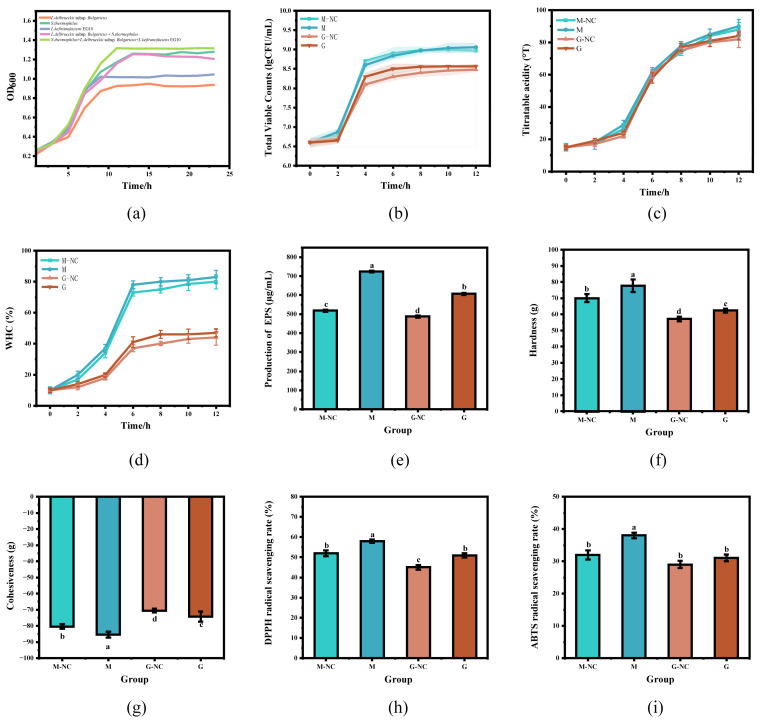
Effects of EG10 co-fermentation on the quality characteristics of fermented milk: Growth curves (**a**); total viable counts (TVC) (**b**); titratable acidity (TC) (**c**); water-holding capacity (WHC) (**d**); production of EPS (**e**); hardness (**f**); cohesiveness (**g**); DPPH radical scavenging ability (**h**); ABTS radical scavenging ability (**i**). Different letters suggest significant difference (*p* < 0.05).

The quality characteristics of fermented milk are presented in Figure 7. Compared with the monoculture, the optical density (OD) values of the three-strain co-culture system significantly increased after the addition of EG10 (Figure 7a), indicating that EG10 promoted the growth of the starter cultures. With increasing fermentation time, both the total viable counts and titratable acidity of all fermented milk samples showed a continuous increase, with fermented cow milk (M) exhibiting higher values than fermented goat milk (G) (Figure 7b,c), which is consistent with the general characteristics of lactic acid fermentation.

Compared with the control groups, the water-holding capacity (WHC) of fermented milk in the co-fermentation groups was significantly improved, and fermented cow milk (M) exhibited a higher WHC than fermented goat milk (G) (Figure 7d). This enhancement may be attributed to the higher viable cell counts in fermented cow milk (M) and the production of EPS by EG10. EPS possess a strong water-binding capacity, which effectively limits whey separation and reduces free water release, thereby improving the WHC of fermented milk [64]. Further analysis revealed that the polysaccharide contents of fermented milk in the co-fermentation groups (724 μg/mL for M and 606 μg/mL for G) were significantly higher than those of the corresponding control groups (518 μg/mL for M-NC and 487 μg/mL for G-NC) (Figure 7e), indicating that co-fermentation with EG10 and conventional starter cultures markedly enhanced polysaccharide accumulation.

Texture profile analysis showed that the hardness and cohesiveness of fermented cow milk (M) in the co-fermentation group were significantly higher than those of the control group (M-NC) and were also significantly higher than those of fermented goat milk (G) (Figure 7f,g). These results suggest that the addition of EG10 contributed to the formation of a more compact gel network structure and enhanced intermolecular interactions within the gel matrix [65].

In addition, fermented cow milk (M) in the co-fermentation group exhibited significantly higher DPPH and ABTS radical scavenging activities than fermented goat milk (G) and the control group (M-NC) (Figure 7h,i), indicating that the incorporation of EG10 significantly enhanced the antioxidant capacity of fermented milk, with a more pronounced effect observed in fermented cow milk. This improvement is mainly associated with the increased production of EPS.

Overall, co-fermentation with EG10 and conventional starter cultures effectively improved the physicochemical properties of fermented milk, including water-holding capacity, textural properties, and antioxidant activity. Under identical fermentation conditions, fermented cow milk exhibited superior quality characteristics compared with fermented goat milk.

## 4. Conclusions

In this study, three high EPS-producing lactic acid bacteria were successfully isolated from kefir grains and identified as *L. plantarum* XZ61, *L. kefiranofaciens* EG10, and *L. kefiri* EG12. All three strains exhibited strong probiotic characteristics, including high survival under acidic (pH 2.5) and bile salt (0.1%, *w*/*v*) conditions, confirming their potential as robust probiotics. After optimization of fermentation parameters, the EPS yield of *L. kefiranofaciens* EG10 reached 539.57 μg/mL, representing the highest production among the isolates.

Structural characterization using HPSEC revealed that the EPS from each strain were heteropolysaccharides composed mainly of mannose (33.38~61.58%), along with glucose, galactose, and uronic acids. These compositional differences influenced their physicochemical properties and bioactivities. Antioxidant assays demonstrated strong ABTS^•+^ and hydroxyl radical scavenging capacities, particularly for EG10-EPS, achieving up to 49.86% and 86.67% inhibition, respectively. Differential scanning calorimetry further confirmed the excellent thermal stability of the EPS, supporting their suitability for use in heat-processed foods.

Importantly, the practical application of the selected EPS-producing strains in yogurt fermentation demonstrated their capacity to effectively participate in milk fermentation and modulate key quality attributes of the final product. The presence of these strains and their in situ–produced EPS contributed to improvements in yogurt texture and structural stability, highlighting the functional relevance of kefir-derived LAB and their EPS within real dairy matrices rather than model systems alone.

Overall, these findings identify kefir-derived LAB as promising sources of multifunctional postbiotic EPS with direct relevance to fermented milk production. In particular, the EPS produced by *L. kefiranofaciens* EG10 combine strong techno-functional properties, including emulsification and flocculation, with notable antioxidant activity and demonstrated performance in yogurt fermentation. This study provides a solid scientific foundation for the development of next-generation synbiotic or functional dairy products, in which EPS-producing LAB and their biopolymers can synergistically enhance both technological quality and health-related value in yogurt and other fermented dairy foods. In addition, LAB-derived EPS and associated metabolites have been reported to exhibit inhibitory effects against common foodborne pathogens such as Escherichia coli and Staphylococcus aureus, as well as certain opportunistic fungi. Therefore, the strains identified in this study may also contribute to improving microbial safety and extending shelf life in fermented dairy products, highlighting their broader relevance to food safety and public health.

## Data Availability

The original contributions presented in the study are included in the article. Further inquiries can be directed to the corresponding authors.
